# KCNK10, a Tandem Pore Domain Potassium Channel, Is a Regulator of Mitotic Clonal Expansion during the Early Stage of Adipocyte Differentiation

**DOI:** 10.3390/ijms151222743

**Published:** 2014-12-09

**Authors:** Makoto Nishizuka, Takahiro Hayashi, Mami Asano, Shigehiro Osada, Masayoshi Imagawa

**Affiliations:** Department of Molecular Biology, Graduate School of Pharmaceutical Sciences, Nagoya City University, 3-1 Tanabe-dori, Mizuho-ku, Nagoya, Aichi 467-8603, Japan; E-Mails: prmakoto@phar.nagoya-cu.ac.jp (M.N.); p052759@phar.nagoya-cu.ac.jp (T.H.); p112711@phar.nagoya-cu.ac.jp (M.A.); osada@phar.nagoya-cu.ac.jp (S.O.)

**Keywords:** KCNK10, tandem pore domain potassium channel, adipocyte differentiation, mitotic clonal expansion, insulin signaling

## Abstract

KCNK10, a member of tandem pore domain potassium channel family, gives rise to leak K^+^ currents. It plays important roles in stabilizing the negative resting membrane potential and in counterbalancing depolarization. We previously demonstrated that *kcnk10* expression is quickly elevated during the early stage of adipogenesis of 3T3-L1 cells and that reduction of *kcnk10* expression inhibits adipocyte differentiation. However, the molecular mechanism of KCNK10 in adipocyte differentiation remains unclear. Here we revealed that *kcnk10* is induced by 3-isobutyl-1-methylxanthine, a cyclic nucleotide phosphodiesterase inhibitor and a potent inducer of adipogenesis, during the early stage of adipocyte differentiation. We also demonstrated that KCNK10 functions as a positive regulator of mitotic clonal expansion (MCE), a necessary process for terminal differentiation. The reduction of *kcnk10* expression repressed the expression levels of CCAAT/enhancer-binding protein β (C/EBPβ) and C/EBPδ as well as the phosphorylation level of Akt during the early phase of adipogenesis. In addition, knockdown of *kcnk10* expression suppressed insulin-induced Akt phosphorylation. These results indicate that KCNK10 contributes to the regulation of MCE through the control of C/EBPβ and C/EBPδ expression and insulin signaling.

## 1. Introduction

Tandem-pore domain potassium (K_2P_) channels give rise to leak K^+^ currents, stabilize the negative resting membrane potential, and counterbalance depolarization [1,2,3,4,5]. K_2P_ channels have two P domains and four transmembrane segments. Fifteen different channels comprise the K_2P_ channel family, which can be divided into six distinct subfamilies, including the tandem of pore domains in a weak inward rectifying K^+^ channel (TWIK), TWIK-related acid-sensitive K^+^ channel (TASK), TWIK-related K^+^ channel (TREK), tandem pore domain halothane inhibited K^+^ channel (THIK), TWIK-related alkaline pH-activated K^+^ channel (TALK), and TWIK-related spinal cord K^+^ channel (TRESK) subfamilies. The TREK subfamily is composed of KCNK2 (also called TREK1), KCNK10 (also called TREK2), and KCNK4 (also called TRAAK). These proteins are activated by physiological and chemical stimulation, such as free fatty acids, membrane stretch, intracellular pH, and volatile anesthetics, and are involved in neuroprotection, anesthesia, and pain perception [1,3,6]. It has been reported that KCNK10 is mainly expressed in the cerebellum, and contributes to a neural background conductance [7]. Recently, Cadaveira-Mosquera *et al.* showed that KCNK10 is crucial for the resting membrane potential in mouse superior cervical ganglion neurons [8]. Furthermore, KCNK10 is involved in the control of neural excitability and spatial learning [9]. The functions of KCNK10 in peripheral tissues are poorly understood, whereas the functions of KCNK10 in the central nervous system have been extensively studied.

Previously, Sato *et al.* [10] mapped the quantitative trait loci (QTL) for intramuscular fat content by conducting a linkage analysis of porcine chromosome 7. The intramuscular fat QTL region in the porcine genome corresponds to mouse chromosome 12 [10]. Comparison of this region with the mouse gene map indicated the presence of 11 genes in this critical region. KCNK10 was identified as one of these 11 genes. Therefore, we examined the role of KCNK10 during adipocyte differentiation using mouse 3T3-L1 preadipocytes. We demonstrated that the expression of *kcnk10* was quickly elevated during the early stage of adipogenesis in 3T3-L1 cells and the reduction of *kcnk10* expression inhibited adipocyte differentiation [11]. Although these results suggested that KCNK10 has a crucial role for adipocyte differentiation, the molecular mechanism of KCNK10 was unclear.

In this paper, we first demonstrate that *kcnk10* expression is strongly induced by 3-isobutyl-1-methylxanthine (IBMX), a potent inducer of adipocyte differentiation. Furthermore, we show that KCNK10 functions as a positive regulator of mitotic clonal expansion (MCE), a process required for terminal differentiation. Reduced of *kcnk10* expression repressed the expression levels of CCAAT/enhancer-binding protein β (C/EBPβ) and C/EBPδ as well as the phosphorylation level of Akt during the early stage of adipogenesis. In addition, knockdown of *kcnk10* expression suppressed insulin-induced Akt phosphorylation. These results indicate that KCNK10 contributes to the regulation of MCE through the control of C/EBPβ and C/EBPδ expression and the insulin signaling pathway.

## 2. Results and Discussion

### 2.1. Results

#### 2.1.1. Effect of Adipogenic Inducers on the Expression of *kcnk10*

**Figure 1:**
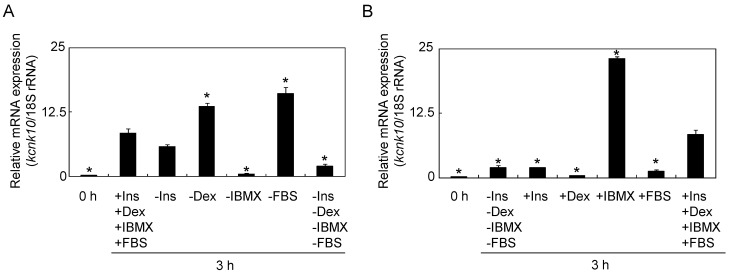
Effect of adipogenic inducers on the *kcnk10* expression. (**A**) The effects of media in which only one inducer was omitted on *kcnk10* expression; and (**B**) The effects of media in which only one inducer was added. In columns labeled “−” the indicated inducer was omitted; in those labeled “+” the indicated inducer was added. Total RNA was prepared from 3T3-L1 cells before induction (0 h) and at 3 h after the addition of various inducers. The expression level of *kcnk10* was normalized to the 18S rRNA expression level, determined by quantitative real time PCR (Q-PCR). The data represent means with standard deviations (*n* = 3). The asterisks indicate significant differences (* *p* < 0.01 *vs.* “+Ins, +Dex, +IBMX, +FBS”).

To differentiate 3T3-L1 preadipocytes into mature adipocytes, a combination of IBMX, dexamethasone (Dex), insulin, and fetal bovine serum (FBS) were added to the medium. To characterize the effects of these inducers, we first examined the expression profiles of *kcnk10* using deprivation media in which only one of the inducers was omitted. As in our previous study, *kcnk10* expression was up-regulated at 3 h after treatment with the medium containing all four inducers. When IBMX was omitted, the induction level of *kcnk10* was drastically and significantly decreased (Figure 1A). In contrast, the expression level of *kcnk10* was significantly increased by the deprivation of Dex or FBS. Next, we examined the levels of *kcnk10* induction in media to which only one inducer was added. The expression of *kcnk10* was slightly increased at 3 h after treatment with a medium to which none of the inducers was added. The treatment of insulin only (“+Ins”), Dex only (“+Dex”), or FBS only (“+FBS”) reduced significantly the *kcnk10* expression compared with “+Ins, +Dex, +IBMX, +FBS”. On the other hand, *kcnk10* expression was markedly and significantly induced by IBMX treatment (Figure 1B). These results demonstrated that IBMX is a major inducer of *kcnk10* expression during adipocyte differentiation.

#### 2.1.2. The Role of KCNK10 on MCE

**Figure 2:**
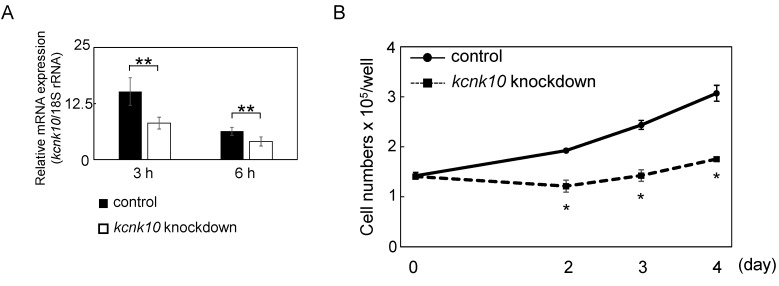
Effect of knockdown of *kcnk10* expression on MCE. (**A**) Knockdown of *kcnk10* expression by short hairpin RNA (shRNA) expression plasmid at 3 and 6 h after induction. Total RNA obtained from 3T3-L1 cells transfected with a shRNA expression plasmid targeting *kcnk10* was subjected to Q-PCR. A scrambled shRNA expression plasmid was used as a control. The level of *kcnk10* expression was normalized to the 18S rRNA expression determined by Q-PCR. The data represent means with standard deviations (*n* = 3). The asterisks indicate significant differences compared with the values for control cells (** *p* < 0.05); and (**B**) At 72 h after transfection, post-confluent *kcnk10* knockdown cells or control cells were induced to differentiate into adipocytes. Cell numbers were determined at various time points using a hemocytometer. The data represent means with standard deviations (*n* = 3). The asterisks indicate significant differences compared with the values for control cells (* *p* < 0.01).

Our previous studies showed that *kcnk10* was expressed transiently within 3 h of induction of adipocyte differentiation [11]. Therefore, we next examined whether KCNK10 was involved in the regulation of MCE, which is observed during the early stage of adipogenesis and is required for adipocyte differentiation. We first determined the expression level of *kcnk10* in 3T3-L1 cells transfected with short hairpin RNA (shRNA) expression plasmid by Q-PCR. As shown in our previous study, in cells transfected with shRNA expression plasmid, the mRNA level of *kcnk10* was reduced by 50% and 40% compared with that in the control cells at 3 and 6 h after induction, respectively (Figure 2A). 3T3-L1 preadipocytes treated with shRNA for *kcnk10* were induced to differentiate, and MCE was assessed by cell counting at different time points using a hemocytometer (Figure 2B). Preadipocytes treated with the control plasmid underwent an almost 3-fold increase in number by Day 4. The cell numbers of preadipocytes treated with shRNA for *kcnk10* were significantly lower than those of control cells at Days 2, 3, and 4. This result suggested that the knockdown of *kcnk10* expression impairs MCE.

#### 2.1.3. Effect of Knockdown of *kcnk10* Expression on the Expression Level of C/EBPβ and C/EBPδ

**Figure 3:**
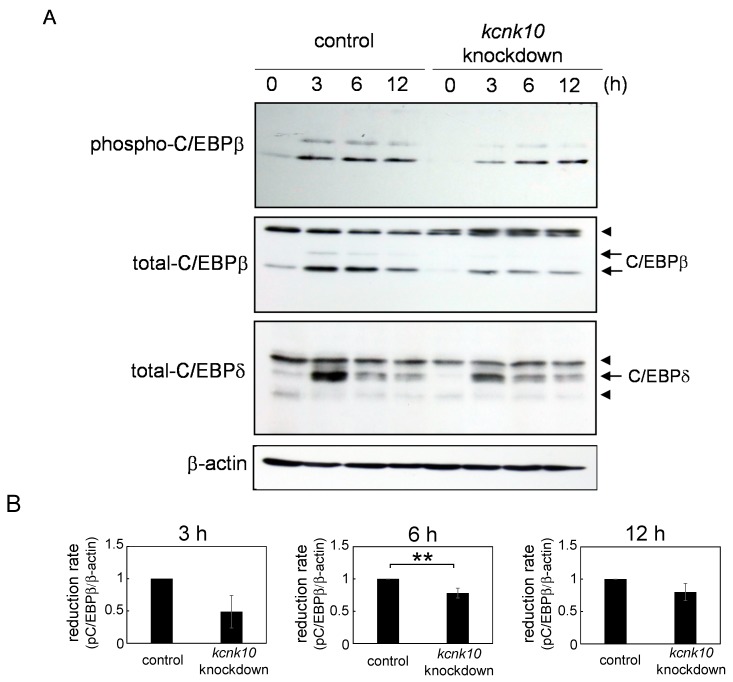

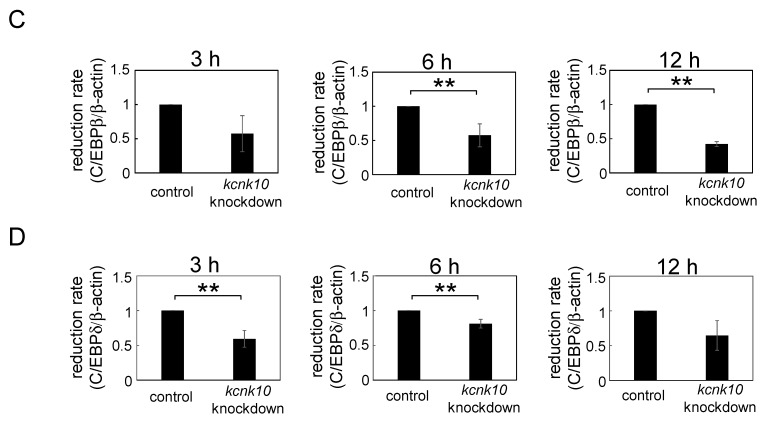
Effect of *kcnk10* knockdown on the phosphorylation and protein levels of C/EBPβ, and the protein level of C/EBPδ. At 72 h after transfection, post-confluent *kcnk10* knockdown cells or control cells were induced to differentiate into adipocytes. Whole-cell lysates were prepared from the cells at various time points after induction and were subjected to Western blot analyses. β-actin was used as a loading control. (**A**) These figures show typical results. Arrowheads show nonspecific bands; (**B**) Quantitation of reduction rate of phospho-C/EBPβ after induction; (**C**) Quantitation of reduction rate of total-C/EBPβ after induction; and (**D**) Quantitation of reduction rate of C/EBPδ after induction. The asterisks indicate significant differences compared with the values for control cells (** *p* < 0.05). All immunoblots obtained from 3 different lots were shown in Supplementary Figures S1–S4.

Tang *et al.* have demonstrated that C/EBPβ is required for MCE during adipocyte differentiation [12]. We found that C/EBPδ is also required for MCE [13]. Therefore, we next examined whether reduction of *kcnk10* expression influences in the expression levels of C/EBPβ and C/EBPδ. Two specific bands were detected by the immunoblots of phospho-C/EBPβ and total C/EBPβ. Since the lower bands were main products as reported previously [14], the lower bands were used for quantitation of phospho-C/EBPβ and total C/EBPβ. As shown in Figure 3, the expression levels of C/EBPβ was significantly decreased in *kcnk10* knockdown cells at 6 and 12 h after induction (Figure 3A,C). Furthermore, a decrease in the phosphorylation level of C/EBPβ was also observed in *kcnk10* knockdown cells (Figure 3A,B). Since the reduction rate of phosphorylation level of C/EBPβ were comparable with that of C/EBPβ protein level, it was thought that the reduction of C/EBPβ phosphorylation by *kcnk10* knockdown might be caused by decrease of expression level of C/EBPβ. The protein level of C/EBPδ was quickly elevated at 3 h after induction and gradually decreased. As shown in Figure 3A,D, the expression level of C/EBPδ was significantly reduced in *kcnk10* knockdown cells at 3 and 6 h after induction. These results showed that KCNK10 has a crucial role in the regulation of C/EBPβ and C/EBPδ expression during the early stage of adipogenesis.

#### 2.1.4. Effect of Knockdown of *kcnk10* Expression on Insulin Signaling Pathway

**Figure 4:**
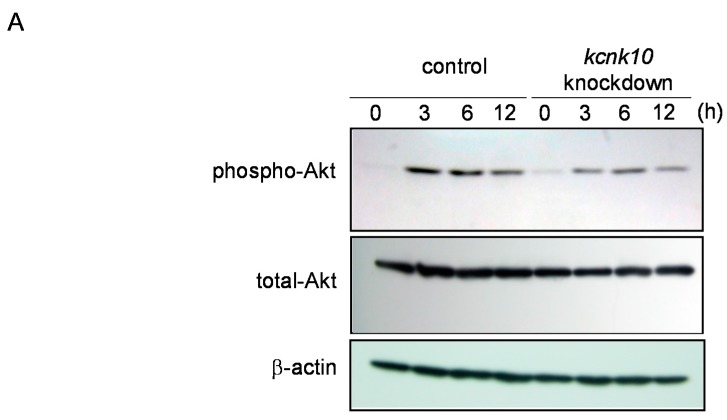

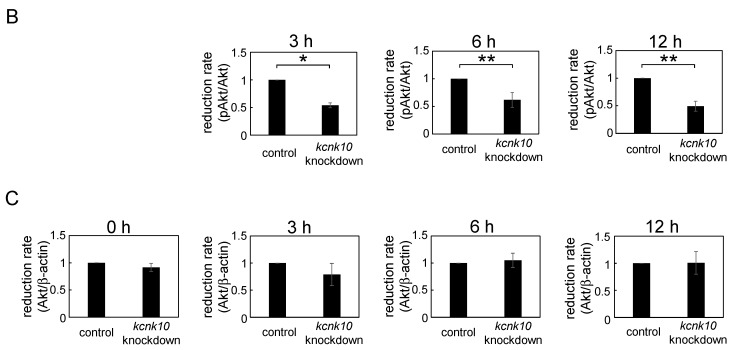
Effect of *kcnk10* knockdown on Akt phosphorylation. At 72 h after transfection, post-confluent *kcnk10* knockdown cells or control cells were induced to differentiate into adipocytes. Whole-cell lysates were prepared at various time points after induction and were subjected to Western blot analyses. (**A**) These figures show typical results; (**B**) Quantitation of reduction rate of phospho-Akt after induction; and (**C**) Quantitation of reduction rate of total-Akt after induction. The asterisks indicate significant differences compared with the values for control cells (* *p* < 0.01, ** *p* < 0.05). All immunoblots obtained from 3 different lots were shown in Supplementary Figures S1–S3.

Insulin is a key regulator of adipocyte differentiation and contributes to the regulation of MCE. For example, Nakae *et al.* revealed that insulin suppresses Foxo1 activity to prevent activation of the cyclin-dependent kinase inhibitor p21 until cell growth has subsided [15]. Furthermore, experiments using a phosphatidylinositol-3 kinase (PI3K) inhibitor showed that the activation of PI3K was required for MCE [16,17]. Therefore, we next examined whether reduction of *kcnk10* expression alters the phosphorylation level of Akt during adipocyte differentiation. The phosphorylation level of Akt at the early stage of adipogenesis was significantly suppressed by the reduction of *kcnk10* expression, whereas the level of total Akt did not differ between *kcnk10* knockdown and control cells (Figure 4).

**Figure 5:**
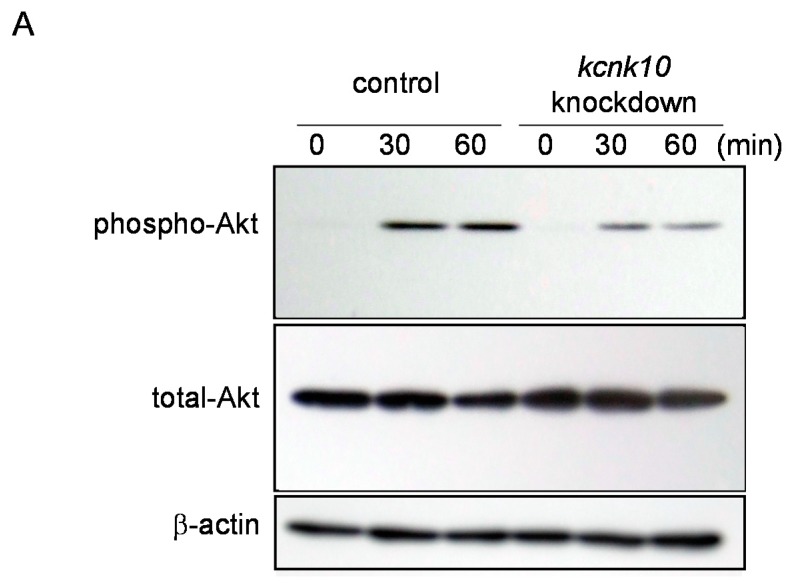

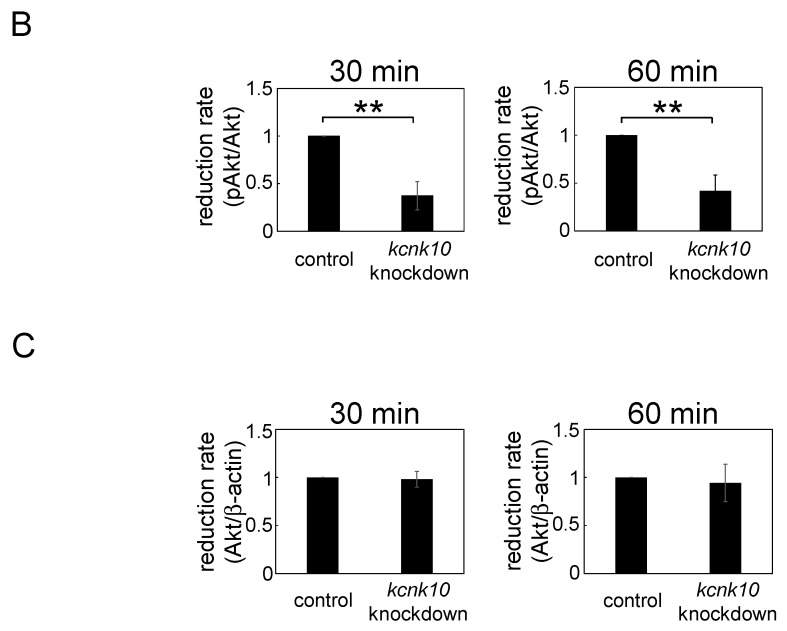
Effect of *kcnk10* knockdown on insulin-induced Akt phosphorylation. *Kcnk10* knockdown cells or control cells were starved for 4 h and treated with 10 nM insulin. Whole-cell lysates were prepared at various time points after treatment and were subjected to Western blot analyses. β-actin was used as a loading control. (**A**) These figures show typical results; (**B**) Quantitation of reduction rate of phospho-Akt after insulin stimulation; and (**C**) Quantitation of reduction rate of total-Akt after insulin stimulation. The asterisks indicate significant differences compared with the values for control cells (** *p* < 0.05). All immunoblots obtained from 3 different lots were shown in Supplementary Figure S5.

Because the adipogenic medium contained IBMX, Dex, FBS, and insulin, we finally investigated whether KCNK10 regulates Akt phosphorylation induced solely by insulin. After transfection, *kcnk10* knockdown and control cells were starved for 4 h, and treated with 10 nM insulin. In control cells, the phosphorylation level of Akt was increased at 30 and 60 min after treatment. On the other hand, Akt phosphorylation in *kcnk10* knockdown cells was clearly and significantly inhibited compared with that in control cells (Figure 5). These results indicate that KCNK10 assumes an important role in the regulation of the insulin signaling pathway during the early stage of adipogenesis.

### 2.2. Discussion

MCE is required for the progression of the adipocyte differentiation program [12,18]. Previous studies demonstrated that C/EBPβ expression occurred concomitant with the entry S phase at the G_1_/S checkpoint [12]. Furthermore, S-phase kinase-associated protein (Skp2) has an important role in targeting the cell-cycle inhibitor p27 for degradation by the 26S proteasome during MCE [19]. We also showed that knockdown of the expression of TC10-like/TC10βLong (*TCL/TC10βL*), regulators of G protein signaling 2 (*RGS2*), factor for adipocyte differentiation 104 (*fad104*), *fad24*, and paternally expressed gene 10 (*peg10*), which are induced during the early phase of adipogenesis, impaired MCE [20,21,22]. Considering these findings, although it is thought that MCE is regulated by many factors in a very complex way, the molecular mechanism of MCE is not fully understood.

In this study, we demonstrated for the first time that KCNK10, a member of tandem pore domain potassium channel family, has a crucial role in the regulation of MCE. Since we previously showed that knockdown of *kcnk10* expression impairs adipocyte differentiation, it would appear that KCNK10 promotes adipogenesis through the control of MCE. Although the expression level of *kcnk10* in knockdown cells was reduced approximately 50% compared with that in control cells at the peak time after induction, the cell growth during MCE was drastically inhibited in *kcnk10* knockdown cells (Figure 2). We used Nucleofector for transfection with high efficiency in 3T3-L1 cells. Therefore, it is likely that the reduction by approximately half of *kcnk10* expression in each cell transfected is enough to cause potent inhibition of MCE. As shown in Figure 3 and Figure 4, the reduction of *kcnk10* expression repressed the expression levels of C/EBPβ and C/EBPδ and suppressed Akt phosphorylation during MCE, implying that the inability to undergo MCE observed in *kcnk10* knockdown cells was accompanied by the down-regulation of C/EBPβ and C/EBPδ expression and/or the insulin signaling pathway. Some earlier studies showed that cAMP-responsive element-binding protein (CREB) has a crucial role in the regulation of C/EBPβ and C/EBPδ expression [23,24]. In addition, MacDougald *et al.* demonstrated that insulin also up-regulated the transcription of C/EBPβ and C/EBPδ genes [25]. Because the knockdown of *kcnk10* drastically decreases the phosphorylation level of Akt, KCNK10 may regulate C/EBPβ and C/EBPδ expression via the insulin signaling pathway. Since we have no information whether KCNK10 regulates Akt phosphorylation directly or not yet, further analyses including co-immunoprecipitation and immunofluorescence is necessary to clarify how KCNK10 regulates the phosphorylation level of Akt and insulin signaling pathway.

KCNK10 contributes to the stability of the resting membrane potential and opposes depolarization and cell excitability in various cells [1,2]. Sundelacruz *et al.* showed that membrane depolarization caused by the addition of 80 mM K^+^ and 10 nM ouabain, a Na^+^/K^+^-ATPase inhibitor, inhibits adipocyte differentiation of human mesenchymal stem cells (hMSCs) [26]. On the other hand, it was reported that membrane depolarization is the trigger for PI3K/Akt activation in endothelial cells [27]. These findings raise the possibility that changes in membrane potential contribute to the regulation of MCE. Research is definitely needed to explore whether KCNK10 regulates membrane potential and whether KCNK10-mediated changes in membrane potential influence the insulin signaling pathway and adipocyte differentiation. In addition, it is required to perform experiments using dominant negative and/or constitutively active form of KCNK10 to clarify whether the channel activity of KCNK10 is necessary to regulate MCE and adipocyte differentiation or not.

Lauritzen *et al.* demonstrated that KCNK10 and KCNK2 shape the actin cytoskeleton independently of their channel activity [28]. Whereas this interesting property was observed only in the TREK subfamily, it seems that other K_2P_ channel subfamilies do not have it [28]. Recently, it was reported that a change in cell shape, which is determined by the dynamics of the actin cytoskeleton during the early stage of adipocyte differentiation, is necessary for the promotion of adipogenesis [29]. Considering these reports, it is possible that KCNK10 regulates MCE and adipogenesis through the control of actin remodeling. It is also necessary to elucidate the relationship between KCNK10 and changes in the actin cytoskeleton during MCE.

Even though “+IBMX” and “+Ins, +Dex, +IBMX, +FBS” contain the same amount of IBMX, the expressin level of *kcnk10* was drastically increased in the treatment of IBMX only (Figure 1B). Since Dex and FBS down-regulated the *kcnk10* expression (Figure 1A), this might be caused by inhibitory effect of Dex and FBS on *kcnk10* expression during adipocyte differentiation.

IBMX increases the intracellur cAMP level, and mainly activates the protein kinase A (PKA) signaling pathway. It is thought that PKA activated by cAMP promotes adipocyte differentiation in synchronization with exchange protein directly activated by cAMP (Epac) [30]. In addition to its role in adipocyte differentiation, some reports indicate that PKA phosphorylates the COOH terminal region of the TREK subfamily and inhibits channel activity [1,2,5]. Furthermore, the phosphorylation of the COOH terminal region of the TREK subfamily by PKA also has a crucial role in actin remodeling, independent of channel activity [28]. As shown in Figure 1, IBMX functions as a potent inducer of *kcnk10* during adipogenesis, suggesting that the PKA signaling pathway increases *kcnk10* expression and regulates the change in membrane potential and/or actin cytoskeleton caused by KCNK10 during the early stage of adipogenesis. To clarify the molecular mechanism of KCNK10 in adipocyte conversion, the phosphorylation level of KCNK10 must also be analyzed in addition to promoter analysis.

In summary, our present study showed that KCNK10 is necessary for the regulation of MCE. Furthermore, KCNK10 plays an important role in controlling the expression levels of C/EBPβ and C/EBPδ as well as the phosphorylation level of Akt during the early stage of adipogenesis. Recently, it was reported that silencing large-conductance Ca^2+^-activated potassium channels significantly reduces adipogenic differentiation in hMSCs [31]. Furthermore, You *et al.* demonstrated that subsets of voltage-gated K^+^ channels, including Kv2.1 and Kv3.3, play an important role in the differentiation of hMCSs into adipocytes [32]. Considering these findings and our present report, it is expected that many potassium channel subfamilies play central roles during adipocyte differentiation. Further analyses of potassium channels, including KCNK10, will help us understand the signaling pathways during adipogenesis.

## 3. Experimental Section

### 3.1. Cell Culture and Differentiation

Mouse 3T3-L1 preadipocyte cells (Sumitomo Dainippon Pharma, Osaka, Japan) were grown to confluence in Dulbecco’s modified Eagle’s medium (DMEM) containing 10% calf serum (Life Technologies, Carlsbad, CA, USA). Two-day postconfluent cells (day 0) were induced to differentiate with DMEM containing 10% FBS (Life Technologies), 1 μM Dex (Sigma, St. Louis, MO, USA), 10 μg/mL insulin (Sigma), and 0.5 mM IBMX (Nacalai Tesque, Kyoto, Japan) for 2 days. Cells were then fed DMEM supplemented with 10% FBS and 5 μg/mL insulin every other day.

### 3.2. RNAi Experiments

The generation of shRNA expression plasmid for *kcnk10* has been described previously [11]. As a negative control, the scrambled fragment 5'-GTAAGATGAGGCAATGGAG-3', which has no similarity to any mRNA listed in GenBank was generated. 3T3-L1 preadipocytes were transfected with 9 μg of shRNA expression plasmid using the Nucleofector kit V (Lonza, Basel, Switzerland). The cells were then plated in 12-well plates. At two days post-confluence, the transfected cells were induced to differentiate into adipocytes using the inducers described above.

### 3.3. Cell Counting

3T3-L1 cells were trypsinized in 12-well plates at various time points and collected by centrifugation. Cell numbers were measured using a hemocytometer.

### 3.4. Q-PCR

Total RNA was extracted with TriPure (Roche, Basel, Switzerland) according to the manufacturer’s instructions. The total RNA was converted to single-stranded cDNA using a random primer and ReverTra Ace (Toyobo, Osaka, Japan), and the cDNA was used as a template for Q-PCR. An ABI PRISM 7000 sequence detection system (Applied Biosystems, Foster City, CA, USA) was used to perform the Q-PCR experiments. The pre-designed primers and probe sets for *kcnk10* and 18S rRNA were obtained from Applied Biosystems. The reaction mixture was prepared using a TaqMan Universal PCR Master Mix (Applied Biosystems) according to the manufacturer’s instructions. The mixture was incubated at 50 °C for 2 min and at 95 °C for 10 min; subsequently, PCR reactions were performed with 40 cycles of 95 °C for 15 s and 60 °C for 1 min. Relative standard curves were generated in each experiment to calculate the input amounts of unknown samples.

### 3.5. Western Blotting

Equal amounts of total protein were resolved using sodium dodecyl sulfate-polyacrylamide gel electrophoresis (SDS/PAGE) and were transferred to a polyvinylidene difluoride membrane, and probed using primary antibodies and secondary antibodies conjugated with horseradish peroxidase (Jackson ImmunoResearch Laboratories, Inc., West Grove, PA, USA). Specific proteins were detected using an enhanced chemiluminescence system (GE Healthcare, Little Chalfont, UK). Primary antibodies recognizing phospho-C/EBPβ (Cell Signaling Technology, Danvers, MA, USA), C/EBPβ (Santa Cruz Biotechnology, Dallas, TX, USA), C/EBPδ (Santa Cruz Biotechnology), phospho-Akt, Akt (Cell Signaling Technology), and β-actin (Sigma) were used. The intensity corresponding to each band was measured by Image J software (http://imagej.nih.gov/ij/).

### 3.6. Statistical Analyses

Data are presented as mean ± standard deviations (SD), and analyzed with ANOVA using Tukey-Kramer HSD test or Student’s *t* test. A *p*-value of <0.05 was considered statistically significant.

## 4. Conclusions

In this study, we demonstrated that the expression of *Kcnk10*, a member of tandem pore domain potassium channel family, was strongly induced by IBMX, an inducer of adipogenesis. Furthermore, we showed that KCNK10 functions as a positive regulator of mitotic clonal expansion (MCE), a necessary process for terminal differentiation and regulates the expression level of C/EBPβ and C/EBPδ during MCE. In addition, KCNK10 regulates the insulin signaling pathway at the early stage of adipogenesis. These results indicate that KCNK10 contributes to the regulation of MCE through the control of C/EBPβ and C/EBPδ expression as well as insulin signaling.

## Supplementary Materials

Supplementary materials can be ound at at http://www.mdpi.com/1422-0067/15/12/22743/s1.
